# Mechanical Properties and Resistance to Acid Corrosion of Polymer Concrete Incorporating Ceramsite, Fly Ash and Glass Fibers

**DOI:** 10.3390/ma12152441

**Published:** 2019-07-31

**Authors:** Yinong Shen, Bo Liu, Jianfu Lv, Manlin Shen

**Affiliations:** 1 Institute of Advanced Engineering Structures and Materials, Zhejiang University, Hangzhou 310058, China; 2 College of Aerospace and Civil Engineering, Harbin Engineering University, Harbin 150001, China; 3 Zhejiang Huian Engineering Quality Inspection Co., Ltd., Hangzhou 310000, China

**Keywords:** polymer concrete, ceramsite, glass fiber, fly ash, sulfuric acid corrosion

## Abstract

A novel polymer concrete (PC) using an aggregate of ceramsite, fly ash and glass fiber was created. Specimens were used in experiments to investigate its anticorrosion properties to determine the viability of its use in flue gas desulfurization (FGD) stacks. The inclusion of ceramsite reduces both the weight and the cost of the material. The effects of ceramsite and glass fiber on the flexural strength and compressive strength of the concrete were investigated. The experimental results showed that ceramsite reduces the flexural strength and the compressive strength of the concrete, but that the glass fiber increases both. Surface resistance to sulfuric acid corrosion and the microstructure of the corroded concrete were investigated. Specimens of the novel PC and the control PC strongly resisted acid corrosion. Although the specimen surfaces deteriorated, the interior structure of the PC was unaffected after 50 days of acid immersion. Processes by which sulfuric acid corrodes PC surfaces were determined.

## 1. Introduction

Increased regulation of fossil fuel use to protect the environment has led to the adoption of flue gas desulfurization (FGD) systems in thermal power plants to treat the sulfur-containing flue gases released by the combustion of fossil fuels [1]. Acid corrosion seriously reduces the service life of FGD stacks [2]. The renovation of existing FGD stacks requires materials that are used as anticorrosion layers. Materials with extremely useful properties, such as rapid setting and corrosion resistance, and suitable mechanical properties have been developed [3,4,5].

One such material is polymer concrete (PC), which is made from dry aggregates and monomers (binders) that undergo polymerization (hardening) [6,7]. Common PC binders are epoxy resin, unsaturated polyester resin and vinyl ester resin [8,9]. Vinyl ester resins are strongly heat resistant and resist acid corrosion, which makes them suitable for FGD stacks [10,11].

The reuse of industrial wastes is an excellent way to make full use of PC to reduce pollution. Recent studies have used industrial wastes as PC aggregates [12]. The aggregate has a significant effect on the mechanical properties and durability of the concrete produced. Kou and Poon [13] investigated the mechanical properties and durability of a PC that they developed using recycled glass and fly ash as the aggregate. Jafari and Vahab [14] investigated the mechanical properties of a PC that used waste tire rubber as an aggregate. Bulut et al. [15] found that plastic electronic waste increased the ductility of a PC. Bartosz et al. [16] investigated the mechanical properties of a PC using recycled glass as an aggregate.

Sludge ceramsite, produced from dried sewage sludge, clay and water glass (liquid sodium silicate) [17,18] is a pure material. Ceramsite has been used as an aggregate [19,20,21] that produces low-weight and relatively cheap concrete. However, the use of ceramsite may decrease both the compressive strength and the flexural strength of the concrete. Close packing is an excellent way of improving the mechanical properties of the concrete by optimizing the aggregate mix [22,23,24].

The studies identified above show that both the flexural strength and the compressive strength of a PC decrease with the use of industrial wastes. Techniques need to be introduced to reduce the effects of weakened mechanical properties. Reis [25] showed that natural fibers can be used as an effective PC reinforcement. Reis and Ferreira [26] evaluated the mechanical properties of a PC containing short glass and carbon fibers, and found that the fibers increased the flexural strength of the PC.

In this study, we developed a novel polymer concrete using two industrial waste products, ceramsite and fly ash. The addition of glass fiber was necessary for the concrete to withstand FGD use. We compared the compressive strengths, flexural strengths and resistance to acid corrosion of a PC produced using a normal-weight aggregate with concrete produced using a lightweight aggregate (ceramsite). The external and internal concrete structures were analyzed by scanning electron microscopy (SEM, FEI, Hillsboro, OR, USA).

## 2. Raw Materials and Experimental Methods

### 2.1. Raw Materials

#### 2.1.1. Resin

**Figure 1:**
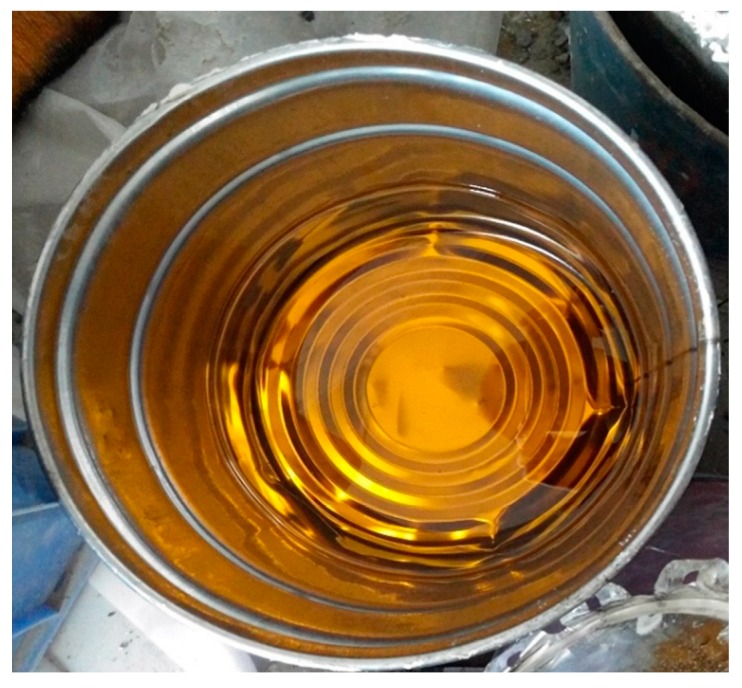
Vinyl ester resin.

**Figure 2:**
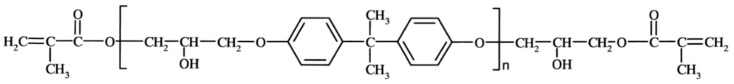
Structure of monomer system.

The characteristics of the vinyl ester resin, purchased from Shanghai Showa Chemicals Co., Ltd. (China), are shown in Figure 1, and the basic resin parameters are given in Table 1. A weight ratio of vinyl ester resin and the hardener (MEKP/cobalt octoate, Shanghai Showa Chemicals Co., Ltd. (Shanghai, China)) was adopted in this study: 100:2:0.8 (vinyl ester resin/MEKP/cobalt octoate). The structure of monomer system is shown in Figure 2 [27].

#### 2.1.2. Ceramsite

**Figure 3:**
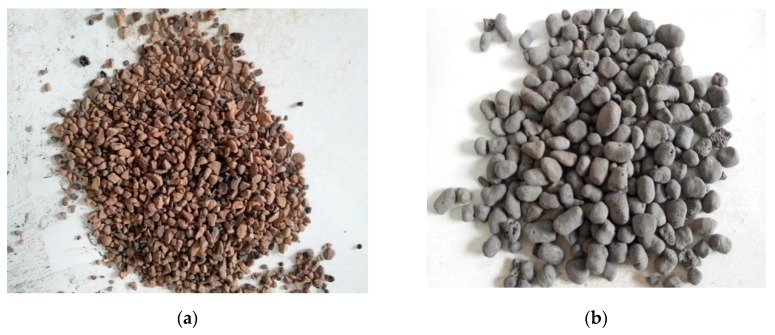
Ceramsite: (**a**) small; (**b**) large.

The ceramsite in this study was divided into small (3–5 mm) and large (10–16 mm). The two kinds of ceramsite are shown in Figure 3. The specific density of small ceramsite was 1.05 g/cm^3^ and the that of large ceramsite was 0.87 g/cm^3^.

#### 2.1.3. Fly Ash

Grade II class F fly ash was used as a fine aggregate in this study. The specific density is 2.44 g/cm^3^ and the chemical composition of fly ash is shown in Table 2.

#### 2.1.4. Glass Fiber

Glass fiber was added to increase the flexural strength of the concrete. The physical properties of glass fiber are given in Table 3.

#### 2.1.5. Sand

Natural river sand with a size of 0.08–2.50 mm was used as the fine aggregate. It had a specific gravity of 2.52 g/cm^3^.

#### 2.1.6. Crushed Stone

Two sizes of crushed stone (3–5 mm and 10–16 mm), with a specific gravity of 2.65 g/cm^3^, were used as aggregates in the normal-weight PC. Crushed stone is made of granite blocks that have been broken up by machines.

### 2.2. Mix Proportions and Specimen Preparation

Close packing theory [22,23,24] was used to determine the mix aggregate proportions of the lightweight PC and the normal-weight PC. Based on the theory, two or three kinds of aggregates were weighed and calculated by volume, packed in an identical container of defined volume and then the mix with the largest equivalent volume that was considered the closest packing mix. The proportions of lightweight PC (LP), lightweight PC containing glass fiber (LPG), normal-weight PC (NP) and normal-weight PC containing glass fiber (NPG) are given in Table 4. The volume of glass fiber was 0.3%.

The concretes were cast into three-point bending molds having dimensions of 40 × 40 × 160 mm. The dimensions of the compressive test specimen molds were 40 × 40 × 40 mm. The resin reached a steady strength after 16 h [16], but in this study, the specimens were cured in the laboratory (20 °C) for 3 d, and then cured at 60 °C for 4 d.

### 2.3. Experimental Methods

#### 2.3.1. Compressive Strength and Flexural Strength Experiments

The three-point bending specimens were tested at 0.2 mm/min, according to Chinese Code GB/T 17671-1999 [28]. The support span length of the specimens was 100 mm. Three samples with a size of 40 × 40 × 160 mm were used to measure the mean flexural strength F (MPa), which was calculated as:

(1) $$F=\frac{1.5NL}{b^{3}}$$

where *N* is the maximum load (kN), *L* is the support span length (mm) and *b* is the side length of the square cross-section (mm).

Three samples, 40 × 40 × 40 mm, were used to measure the mean compressive strength P (MPa), calculated as:

(2) $$P=\frac{N}{A}$$

where N is the maximum load (kN) and A is the cross-sectional area of compression (40 × 40 mm).

#### 2.3.2. Acid Corrosion Experiment

Corrosion in FGD stacks was simulated at 60 °C. To evaluate the resistance to acid corrosion of the PC, the LPG and NPG specimens were immersed in one of three acid solutions: sulfuric acid pH 0.5, sulfuric acid pH 1.0 and sulfuric acid pH 2.0. The experimental procedure followed was that described by Shi [29,30,31]. Concentrated acid was added manually each day to maintain the pH value. The compressive and flexural strengths of the specimens were tested after 10, 20, 30, 40, and 50 d.

#### 2.3.3. Microstructure

**Figure 4:**
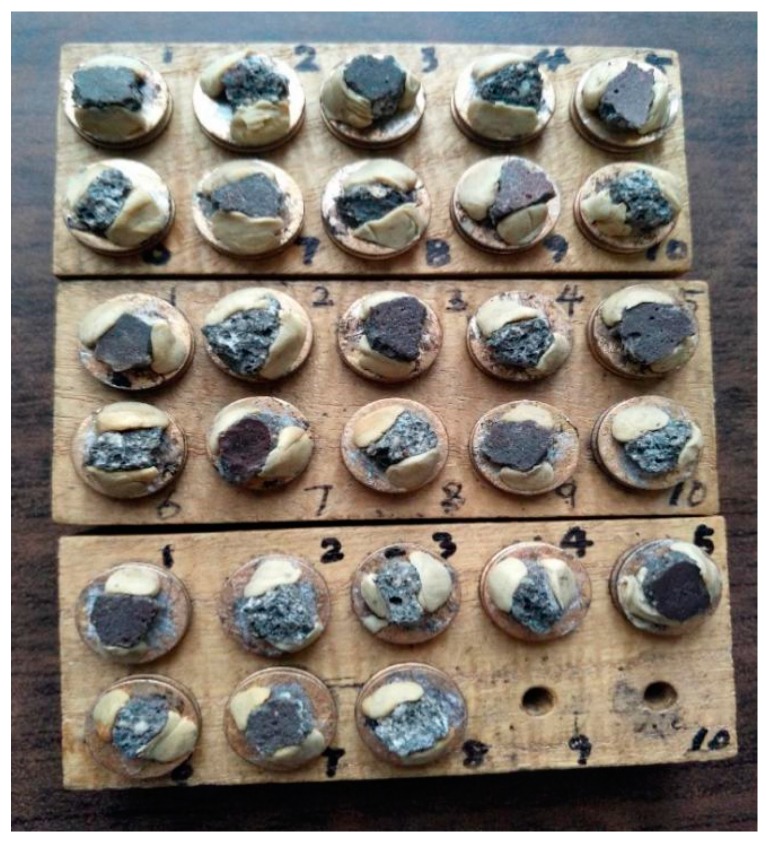
Crushed specimens immersed for 0 d, 30 d, and 50 d polymer concrete (PC).

The microstructure of the concrete was characterized using 0 d, 30 d, and 50 d specimens that were crushed to be observed in detail (Figure 4).

## 3. Results and Discussion

### 3.1. Compressive Strength and Flexural Strength

**Figure 5:**
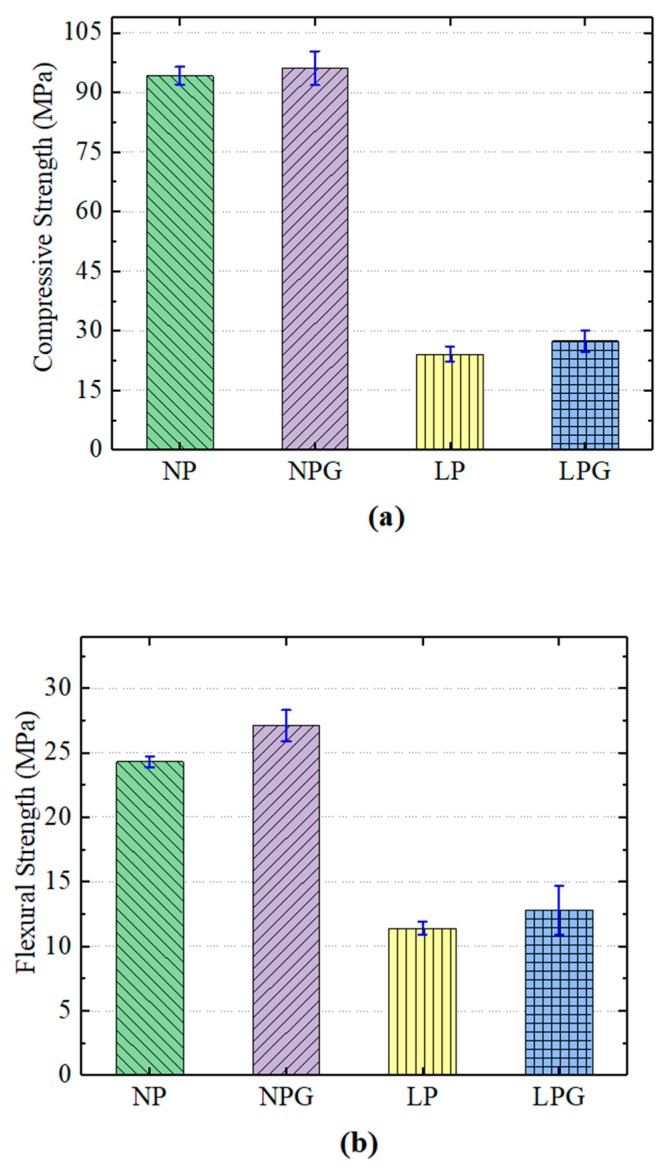
Mechanical properties of the PC specimens: (**a**) compressive strength; (**b**) flexural strength.

Figure 5 shows the compressive strength and the flexural strength of the PC specimens used in the experiments. The figure shows that the replacement of ceramsite significantly decreased both the compressive and the flexural strength. The strengths of the lightweight PC were only about half those of the normal-weight PC because the mechanical properties of ceramsite are worse than those of crushed stones. Zhuang et al. [19] found that the mechanical properties of ceramsite cement concrete were less than those of normal-weight aggregate cement concrete. However, the density of lightweight PC (1.87 g/cm^3^) is almost 70% of that of normal-weight PC (2.69 g/cm^3^). The replacement of ceramsite is an effective method for reduction of PC weight. Moreover, the compressive and flexural strengths of lightweight PC met the engineering requirements for the anti-corrosion layer of FDG stacks, because the anti-corrosion layer only needs to bear its own weight [32]. Furthermore, due to the fact that ceramsite is made from sludge, using ceramsite as an aggregate in PC is a feasible way to recycle the pollution wastes.

The incorporation of glass fiber slightly increased the compressive strength and the flexural strength of both the lightweight PC and the normal-weight PC. The compressive strength of the lightweight PC specimens increased by 2% and their flexural strength increased by 11% compared to the normal-weight PC specimens. This could be due to the fiber bridging [33], whereby the glass fiber increases the compressive strength and the flexural strength by constraining the development of any crack caused by loading. Moreover, the improvement in compressive and flexural strengths can also be attributed to the minimization of crack driving force, which is associated with the difference of the microstructure in the crack arrested zone under static rates of loading [34,35]. Similar strength increases were observed in samples of normal-weight PC, which indicated that ceramsite had little effect on the glass fiber reinforcement of the PC.

It should be noted that the range of error for the PC containing glass fiber was greater than that of the PC without fiber. A reason for this is that the additional microcracks may be related to the poor fiber and matrix bonding, or the poor adhesion between filaments within fiber bundles [33]. This adverse effect makes the degree of the effect of fiber reinforcement uncertain.

### 3.2. Acid Corrosion Test

**Figure 6:**
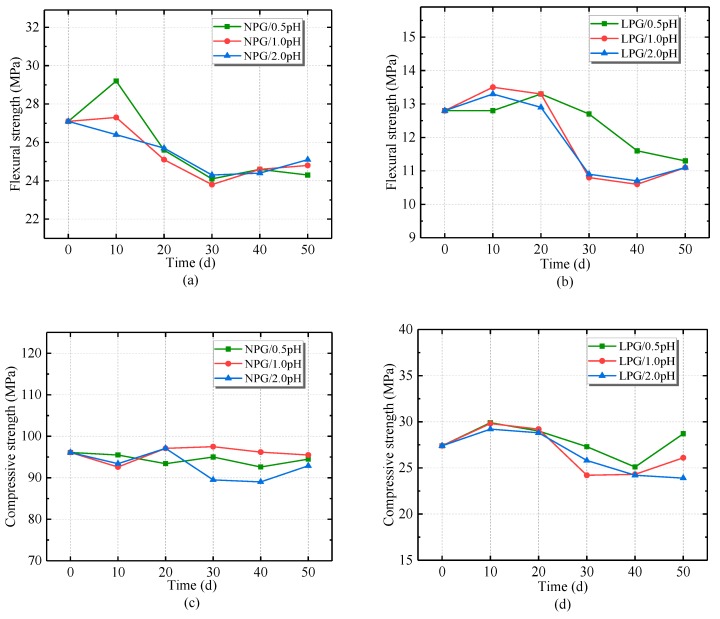
The results of the acid corrosion experiment: (**a**) flexural strength of normal-weight PC containing glass fiber (NPG); (**b**) flexural strength of lightweight PC containing glass fiber (LPG); (**c**) compressive strength of NPG; (**d**) compressive strength of LPG.

Figure 6 shows the results of the acid corrosion experiment. The results show that both types of PC had excellent resistance to acid corrosion.

Figure 6a,b show the flexural strength of the PC specimens. There was a slight decrease in flexural strength over time, which agrees with the results of Gorninski [9], who showed that a PC had great flexural strength and that the addition of fly ash increased the flexural strength. Similar resistance to all levels of acid corrosion was shown by the PC containing different aggregate types. The initial flexural strength values of NPG and LPG were 27.1 MPa and 12.8 MPa. After 50 d exposure to acid, the values of NPG decreased to 24.3 MPa (pH 0.5), 24.8 MPa (pH 1.0) and 25.1 MPa (pH 2.0). The values for LPG decreased to 11.3 MPa (pH 0.5), 11.1 MPa (pH 1.0) and 11.1 MPa (pH 2.0). Norwood [36] found that decreased strength in a PC was due to an increase in porosity, which weakened the bond between the aggregate and the polymer matrix. The flexural strength of LPG fluctuated slightly; it was stable for the first 20 days and then decreased. Figure 6c,d show that the compressive strength of the PC specimens did not vary noticeably over the 50 d acid corrosion experiment. The initial compressive strength of normal-weight PC was 96.1 MPa. After immersion in pH 0.5, pH 1.0 or pH 2.0 sulfuric acid for 50 d, the compressive strength values changed to 94.5 MPa, 95.5 MPa and 92.9 MPa. During the acid immersion, the compressive strengths fluctuated within the allowable range of error. The results for the compressive strengths of LPG specimens were similar.

However, it could be seen that the flexural strengths of NPG and LPG, and the compressive strength of LPG, had a tiny increase after 10 d immersion in the different acid solutions. This could be due to the experimental error or a reduction in the cross-sectional area of the specimen, which reduced the size of the specimens [37]. Because of the size effect, the compressive and flexural strengths of PC specimens might slightly increase after slight corrosion. Further investigation of the microstructure is needed to determine the cause for these increases. The surface of the lightweight PC could have been affected more by acid than the normal-weight PC. Additionally, the increase of compressive strength of LPG at 50 days compared to 40 days was observed. A possible explanation is that some corrosion products filled some pores in the surface zone of the lightweight PC, further increasing the compressive strength of the specimens after a long period of corrosion.

The results showed that both the normal-weight PC and the lightweight PC maintained their compressive strength when acid corrosion in FDG stacks was simulated. The mean flexural strengths of both LPG and NPG decreased by about 15%, which is similar to the results presented by Gorninski et al. [9]. They observed flexural strength losses of 17% in the samples of PC immersed in 5% sulfuric acid, whereas the flexural strength of cement concrete had a 29.2% loss after sulfuric acid [9]. Besides the excellent acid corrosion resistance due to the resin, the fly ash content also had an important effect on the PC [9]. The high fly ash content decreased the number and size of pores in the aggregate, thus reducing diffusion of the sulfuric acid.

### 3.3. Microstructure

#### SEM Analysis

**Figure 7:**
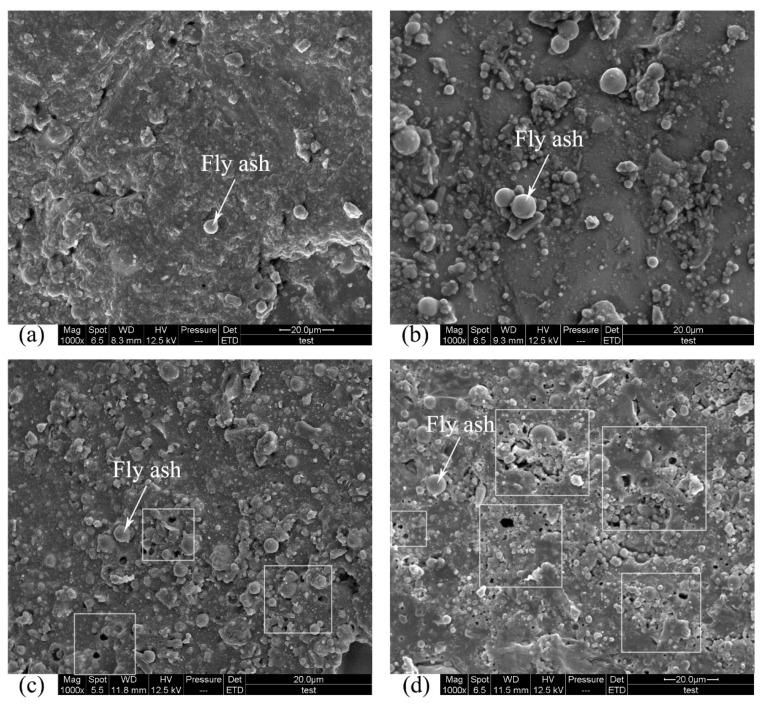
Scanning electron microscopy (SEM) images of the surfaces of lightweight PC specimens after 50 d in sulfuric acid: (**a**) control specimen; (**b**) in pH 2.0 acid; (**c**) in pH 1.0 acid; (**d**) in pH 0.5 acid.

Figure 7 shows the SEM images of the surfaces of lightweight PC specimens that were exposed to 50 d acid corrosion. Fly ash can be seen on the surface of all specimens. It can clearly be seen that the number of fly ash particles on the surface increased as the pH increased. This occurred because the sulfuric acid corroded the PC surface, reducing the cross-sectional area of the specimen and exposing surficial fly ash particles. In comparison with the control specimen (Figure 7a), there were more particles attached to the surface of the specimen immersed in pH 2.0 sulfuric acid. Figure 7c shows that the specimen immersed in pH 1.0 sulfuric acid had more surficial fly ash particles than the specimen immersed in pH 2.0 sulfuric acid, and that some pores were formed on the surface. Increased porosity indicates a lower chemical resistance, which increases the diffusion of corrosive agents [9]. It can be concluded that in pH 1.0 sulfuric acid, lightweight PC forms pores on the surface, reducing chemical resistance, whereas in pH 2.0 sulfuric acid, this phenomenon did not occur during 50 d acid exposure. There was no clear difference in flexural strength or compressive strength between LPG immersed in pH 2.0 and pH 1.0 sulfuric acid. This result indicates that the formation of pores on the surface has little impact on the flexural strength or the compressive strength. However, the formation of pores may have led to further deterioration in the PC, in which case the mechanical properties of PC could lessen. The surface of the specimen immersed in pH 0.5 sulfuric acid (Figure 7d) was worse than that immersed in pH 1.0 sulfuric acid.

**Figure 8:**
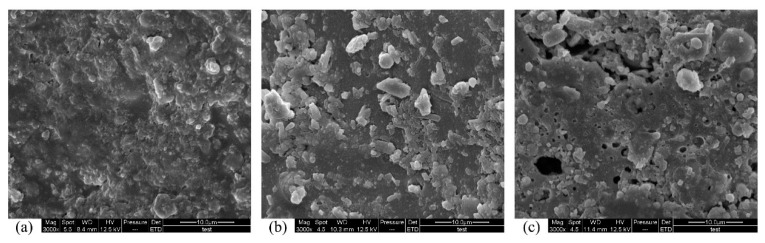
SEM images of surface of lightweight aggregate PC specimens immersed in pH 0.5 sulfuric acid solution: (**a**) 0 d immersion; (**b**) 30 d immersion; (**c**) 50 d immersion.

Differences between the surfaces of specimens immersed in pH 0.5 sulfuric acid for different periods can be observed in Figure 8. By comparing Figure 8a with Figure 8b, the surface of the specimen became roughened, and a number of irregular particles adhered to it. This is due to the fact that sulfuric acid corrodes the surface of the resin matrix and exposes particles in the matrix. In addition, pores appeared on the surfaces of the specimens. Figure 8 shows the process of corrosion on the surface of the lightweight PC specimen. The sulfuric acid reduced the cross-sectional area of the specimens, increased the porosity and extended the pores. The reduction of the cross-sectional area of the specimens might be behind the strength increase of PC specimens in the early stages of corrosion observed in Figure 6.

Although honeycomb and continuous pores were found on the surface, in this case, there was no significant change in the compressive strength or flexural strength compared with corresponding specimens immersed in different sulfuric acids. One reason for this may be that the pores formed on the surface did not extend into the internal matrix. Another reason may be that these pores became filled by some corrosion products, leading to the strength increase after 50 days’ corrosion.

**Figure 9:**
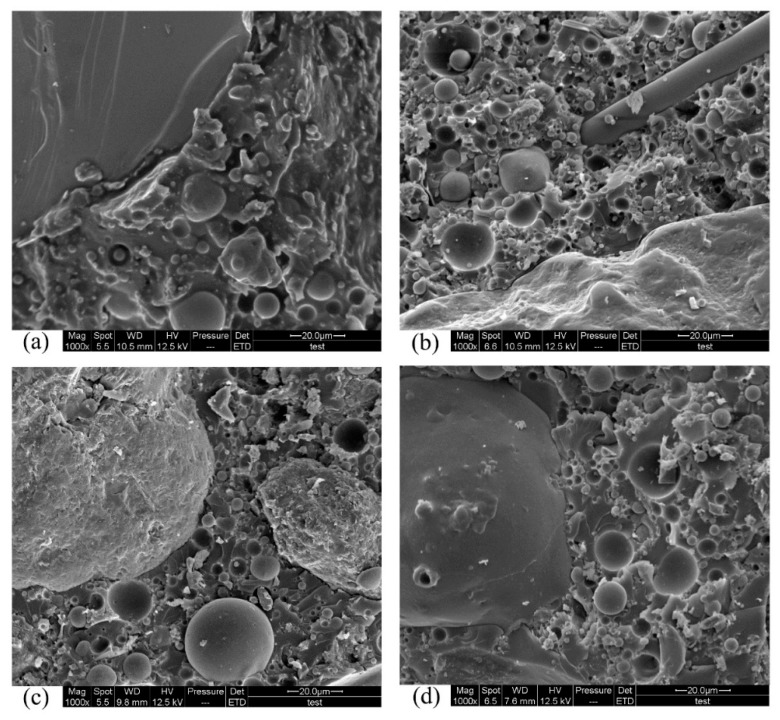
SEM images of the interior of specimens immersed in pH 0.5 acid solution: (**a**) uncorroded NPG; (**b**) corroded NPG; (**c**) uncorroded LPG; (**d**) corroded LPG.

**Figure 10:**
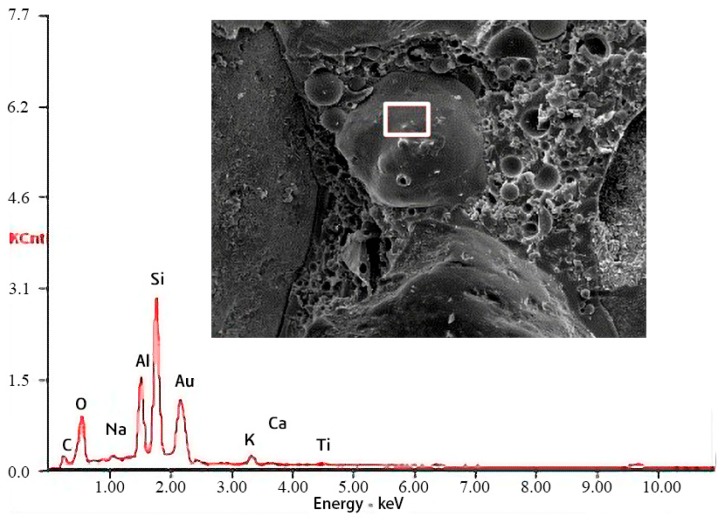
Energy-dispersive X-ray spectroscopy (EDS) analysis corresponding to the SEM images in Figure 9.

Figure 9 shows the internal features of the PC. It can be seen that for both normal-weight PC and lightweight PC, the internal structure of the corroded specimens was as compact as that of the uncorroded specimens. A large irregular particle can be seen at the left of Figure 9d. Energy-dispersive X-ray spectroscopy (EDS, AMETEK, Berwyn, PA, USA) was used for preliminary analysis to confirm that this particle was not produced by corrosion. The corresponding EDS results for the images in Figure 9 are shown in Figure 10, which shows that this particle was not a corrosion product because of a lack of sulfur. Thus, during 50 d acid immersion, the internal structure of PC did not deteriorate. This result is consistent with the preceding results of the compressive strength and flexural strength experiments.

**Figure 11:**
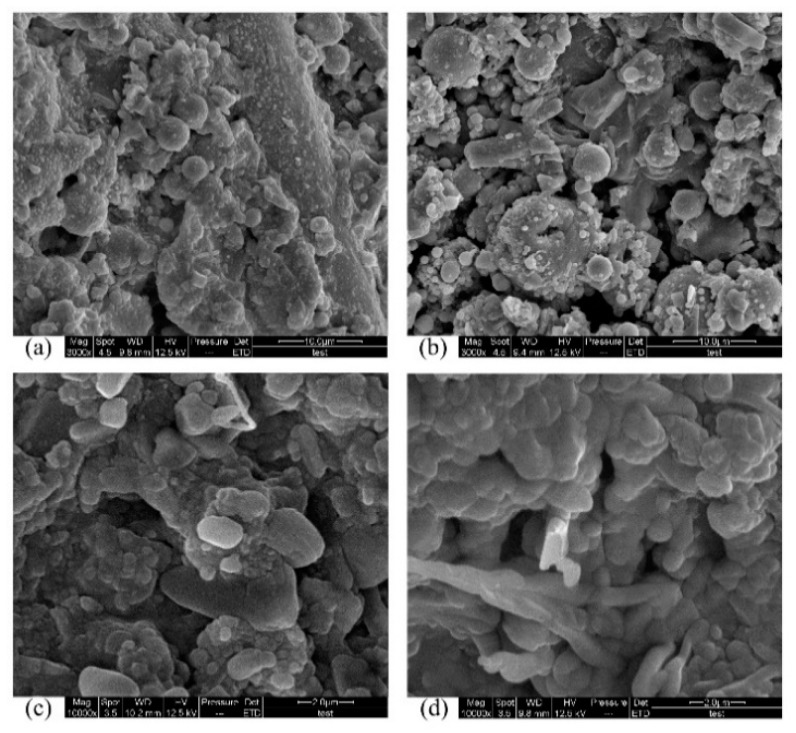
Surfaces of NPG, (**a**) and (**c**), and LPG, (**b**) and (**d**), that were immersed in pH 0.5 acid solution.

**Figure 12:**
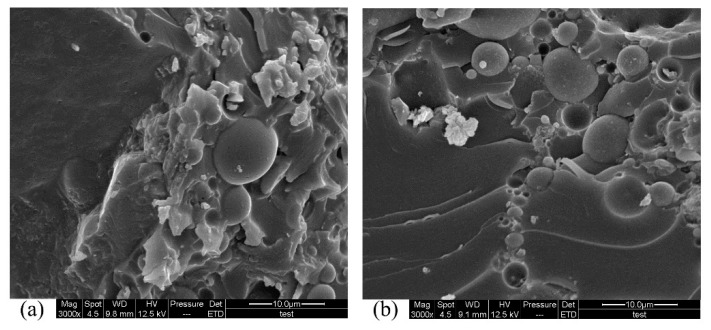
Interiors of NPG, (**a**) and (**c**), and LPG, (**b**) and (**d**), that were immersed in pH 0.5 acid solution.

Figure 11 shows the comparison between NPG and LPG surfaces after 50 d immersion in pH 0.5 sulfuric acid. Both normal-weight PC and lightweight PC displayed similar degrees of acid corrosion. The surfaces of both specimens were rough. There were also pores on the surfaces. It can be seen from the interiors (Figure 12) that the aggregate and matrix are well bonded.

## 4. Conclusions

We developed a novel PC, containing ceramsite, fly ash and glass fiber, which could be applied to the interiors of FGD stacks.

The flexural strength and compressive strength of normal-weight aggregate PC and lightweight aggregate PC were investigated. Although the former performed better, the latter weighed less and had a positive effect on the environment. In addition, the flexural strength and compressive strength of the lightweight PC meet engineering requirements for the anti-corrosion layer of FDG stacks, because the anti-corrosion layer only needs to bear its own weight. Both PCs performed excellently when immersed in sulfuric acid to simulate acid corrosion: the compressive strength of specimens after 50 d immersion was similar to the compressive strength of the control, while the flexural strength decreased slightly. Tiny increases were found in the strengths of PC specimens after 10 d and 50 d immersion. It was surmised that the size effect due to cross-sectional area reduction by immersion caused the increase after 10 d immersion, and that some pores might be filled by corrosion products, causing the increase after 50 d immersion.

SEM and EDS analysis showed that PC strongly resists sulfuric acid corrosion. Immersion in sulfuric acid roughened the surfaces of the specimens and increasingly created pores on the surface. The speed of this process is related to the pH value. However, the interiors of the specimens were unaffected by the acid.

## Figures and Tables

**Table 1:** Basic resin parameters.

Parameter	Value
Specific weight	1.08 g/mL
Brookfield viscosity at 25 °C	325 mPa·s
Styrene content	33%

**Table 2:** The chemical composition of fly ash.

Compositions	Fly Ash (wt%)
CaO	2.6
SiO_2_	63.15
Al_2_O_3_	24.61
Fe_2_O_3_	3.74
MgO	1.64
K_2_O	1.71
Na_2_O	0.58
Alkali content	1.35

**Table 3:** Physical properties of the glass fiber.

Fiber	Glass Fiber
Diameter	15 μm
Length	3 mm
Density	2.54 g/cm^3^
Modulus of elasticity	30.0 GPa
Tensile strength	1510 MPa
Elongation at break	5.4%

**Table 4:** Mix proportions of the concrete (kg/m^3^).

Samples	LP	LPG	NP	NPG
Resin binders	250	250	250	250
Sand	843	843	847	847
Fly ash	307	307	309	309
Large crushed stone	0	0	650	650
Small crushed stone	0	0	633	633
Large ceramsite	223	223	0	0
Small ceramsite	242	242	0	0
Glass fiber	0	7.62	0	7.62

## References

[1] Córdoba P. (2015). Status of Flue Gas Desulphurisation (FGD) systems from coal-fired power plants: Overview of the physic-chemical control processes of wet limestone FGDs. Fuel.

[2] Chen B., Li C. (2007). Study on Stack Corrosion and Anticorrosion after FGD for Coal-fired Units. Power Syst. Eng..

[3] Hong J.H., Lee S.H., Kim J.G., Yoon J.B. (2012). Corrosion behaviour of copper containing low alloy steels in sulphuric acid. Corros. Sci..

[4] Wang L., Gao Y., Xu T., Xue Q. (2006). Corrosion resistance and lubricated sliding wear behaviour of novel Ni–P graded alloys as an alternative to hard Cr deposits. Appl. Surf. Sci..

[5] Yukun Z. (1997). Corrosion of Flue Gas Desulfurization System and its Prevention. Environ. Prot. Chem. Ind..

[6] Gorninski J.P., Dal Molin D.C., Kazmierczak C.S. (2004). Study of the modulus of elasticity of polymer concrete compounds and comparative assessment of polymer concrete and portland cement concrete. Cem. Concr. Res..

[7] Natarajan S., Pillai N.N., Murugan S. (2019). Experimental Investigations on the Properties of Epoxy-Resin-Bonded Cement Concrete Containing Sea Sand for Use in Unreinforced Concrete Applications. Materials.

[8] Vipulanandan C., Paul E. (1993). Characterization of Polyester Polymer and Polymer Concrete. J. Mater. Civ. Eng..

[9] Gorninski J.P., Dal Molin D.C., Kazmierczak C.S. (2007). Strength degradation of polymer concrete in acidic environments. Cem. Concr. Compos..

[10] Kelley D. (2007). The use of FRP in FGD applications. Reinf. Plast..

[11] Han C.D., Lem K.W. (2010). Chemorheology of thermosetting resins. IV. The chemorheology and curing kinetics of vinyl ester resin. J. Appl. Polym. Sci..

[12] Sosoi G., Barbuta M., Serbanoiu A.A., Babor D., Burlacu A. (2018). Wastes as aggregate substitution in polymer concrete. Procedia Manuf..

[13] Kou S.C., Poon C.S. (2013). A novel polymer concrete made with recycled glass aggregates, fly ash and metakaolin. Constr. Build. Mater..

[14] Jafari K., Toufigh V. (2017). Experimental and analytical evaluation of rubberized polymer concrete. Constr. Build. Mater..

[15] Bulut H.A., Şahin R. (2017). A study on mechanical properties of polymer concrete containing electronic plastic waste. Compos. Struct..

[16] Zegardło B., Szeląg M., Ogrodnik P., Bombik A. (2018). Physico-Mechanical Properties and Microstructure of Polymer Concrete with Recycled Glass Aggregate. Materials.

[17] Xu G.R., Zou J.L., Li G.B. (2008). Effect of sintering temperature on the characteristics of sludge ceramsite. J. Hazard. Mater..

[18] Zhao D., Wang F., Liu P., Yang L., Hu S., Zhang W. (2017). Preparation, Physicochemical Properties, and Long-Term Performance of Photocatalytic Ceramsite Sand in Cementitious Materials. Appl. Sci..

[19] Zhuang Y.Z., Chen C.Y., Ji T. (2013). Effect of shale ceramsite type on the tensile creep of lightweight aggregate concrete. Constr. Build. Mater..

[20] Jiao Z., Wang Y., Zheng W., Huang W., Zhou X. (2018). Use of Industrial Waste Slag in Alkali-Activated Slag Ceramsite Concrete Hollow Blocks. Appl. Sci..

[21] Chandra S., Berntsson L. (2002). Lightweight Aggregate Concrete, Science, technology and Applications. Concrete.

[22] Mehta P.K., Aïtcin P.C. (1990). Principles Underlying Production of High-Performance Concrete. Cem. Concr. Aggreg..

[23] Per Goltermann V.J.A.L. (1997). Packing of Aggregates: An Alternative Tool to Determine the Optimal Aggregate. Mixed Mater. J..

[24] Shashidhar N., Gopalakrishnan K. (2011). Evaluating the aggregate structure in hot-mix asphalt using three-dimensional computer modeling and particle packing simulations. Can. J. Civ. Eng..

[25] Reis J.M.L. (2006). Fracture and flexural characterization of natural fiber-reinforced polymer concrete. Constr. Build. Mater..

[26] Reis J.M.L., Ferreira A.J.M. (2004). Assessment of fracture properties of epoxy polymer concrete reinforced with short carbon and glass fibers. Constr. Build. Mater..

[27] Dean K., Cook W.D., Zipper M.D., Burchill P. (2001). Curing behaviour of IPNs formed from model VERs and epoxy systems I amine cured epoxy. Polymer.

[28] Supervision T. (1999). Method of Testing Cements-Determination of Strength, in Chinese Code GB/T 17671–1999.

[29] Shi C., Stegemann J.A. (2000). Acid corrosion resistance of different cementing materials. Cem. Concr. Res..

[30] Vincke E., Verstichel S., Monteny J., Verstraete W. (1999). A new test procedure for biogenic sulfuric acid corrosion of concrete. Biodegradation.

[31] Aydın S., Yazıcı H., Yiğiter H., Baradan B. (2007). Sulfuric acid resistance of high-volume fly ash concrete. Build. Environ..

[32] Zhang Y., Yu P., Pan F., He Y. (2018). The synergistic effect of AFt enhancement and expansion in Portland cement-aluminate cement-FGD gypsum composite cementitious system. Constr. Build. Mater..

[33] Xu S.L., Cai X.R. (2010). Experimental Study and Theoretical Models on Compressive Properties of Ultrahigh Toughness Cementitious Composites. J. Mater. Civ. Eng..

[34] Dutta A., Vanderklok A., Tekalur S.A. (2012). High strain rate mechanical behavior of seashell-mimetic composites: Analytical model formulation and validation. Mech. Mater..

[35] Dutta A., Tekalur S.A. (2014). Crack tortuousity in the nacreous layer—Topological dependence and biomimetic design guideline. Int. J. Solids Struct..

[36] Ls N. Polyester resins in corrosive environments. Proceedings of the Pipecon International Conference on Large Diameter Glass Reinforced Plastic Pipes.

[37] Bažant Z.P. (1999). Size effect on structural strength: A review. Arch. Appl. Mech..

